# Neddylation pattern indicates tumor microenvironment characterization and predicts prognosis in lung adenocarcinoma

**DOI:** 10.3389/fcell.2022.979262

**Published:** 2022-09-13

**Authors:** Yuan Cui, Zhike Chen, Bin Pan, Tong Chen, Hao Ding, Qifan Li, Li Wan, Gaomeng Luo, Lang Sun, Cheng Ding, Jian Yang, Xin Tong, Jun Zhao

**Affiliations:** ^1^ Department of Thoracic Surgery, The First Affiliated Hospital of Soochow University, Suzhou, China; ^2^ Institute of Thoracic Surgery, The First Affiliated Hospital of Soochow University, Suzhou, China; ^3^ Department of Orthopaedics, The First Affiliated Hospital of Nanjing Medical University, Nanjing, Jiangsu, China; ^4^ Soochow University Laboratory of Cancer Molecular Genetics, Medical College of Soochow University, Suzhou, China; ^5^ Department of Cardiovascular Surgery, The First Affiliated Hospital of Soochow University, Suzhou, China

**Keywords:** lung adenocarcinoma, neddylation, tumor microenvironment, prognostic signature, therapeutic response

## Abstract

**Background:** Lung adenocarcinoma (LUAD) is the most common type of lung cancer with a complex tumor microenvironment. Neddylation, as a type of post-translational modification, plays a vital role in the development of LUAD. To date, no study has explored the potential of neddylation-associated genes for LUAD classification, prognosis prediction, and treatment response evaluation.

**Methods:** Seventy-six neddylation-associated prognostic genes were identified by Univariate Cox analysis. Patients with LUAD were classified into two patterns based on unsupervised consensus clustering analysis. In addition, a 10-gene prognostic signature was constructed using LASSO-Cox and a multivariate stepwise regression approach.

**Results:** Substantial differences were observed between the two patterns of LUAD in terms of prognosis. Compared with neddylation cluster2, neddylation cluster1 exhibited low levels of immune infiltration that promote tumor progression. Additionally, the neddylation-related risk score correlated with clinical parameters and it can be a good predictor of patient outcomes, gene mutation levels, and chemotherapeutic responses.

**Conclusion:** Neddylation patterns can distinguish tumor microenvironment and prognosis in patients with LUAD. Prognostic signatures based on neddylation-associated genes can predict patient outcomes and guide personalized treatment.

## Introduction

Lung cancer is a malignant tumor with high morbidity and mortality worldwide (Ferlay et al., 2018; Cao et al., 2020). Histological subtypes of lung cancer can be classified into small cell lung cancer (SCLC) and non-small cell lung cancer (NSCLC). NSCLC can also be divided into lung squamous cell carcinoma (LUSC), lung adenocarcinoma (LUAD), and lung large cell carcinoma (Denisenko et al., 2018). Among them, LUAD is the most common histological subtype and patients with advanced LUAD often have poor prognoses (Hirsch et al., 2017). In addition, LUAD exhibits strong heterogeneity due to the complex tumor microenvironment (TME) and gene mutations (Wang et al., 2018; Jia et al., 2022). Therefore, early risk stratification and individualized treatment have a realistic value for patients with LUAD.

The pathogenesis of LUAD is complex and diverse. In recent years, the role of post-translational modification (PTM) in LUAD has been extensively studied (Park et al., 2020; Bajbouj et al., 2021; De et al., 2021). PTMs can affect the occurrence and development of LUAD by altering target protein activity, protein stability, protein interaction, and intracellular distribution (Stram and Payne, 2016). To date, more than 450 unique protein modifications have been identified (Chen et al., 2020). Like ubiquitination, neddylation can be divided into three steps. First, the neddylation E1 activating enzyme (NAE; a dimer of NAE1 and UBA3) activates the Neural Precursor Cell Expressed Developmentally Downregulated Protein 8 (NEDD8). The NEDD8 is then transferred to the neddylation E2 binding enzyme (UBE2M or UBE2F), and finally to the lysine residues of the target protein in the presence of neddylation E3 ligase which usually contains the cullin structure (Pellegrino et al., 2022). PTMs are involved in the activation of oncogenes, inactivation of tumor suppressor genes, and continuous activation of associated signaling pathways (Perkins, 2006; Han et al., 2018; Chen et al., 2020). Additionally, studies also have shown that many tumor-associated proteins have been reported to be neddylated, but the specific role of neddylation is unclear (Zhao et al., 2014).

The development of cancer is not only related to the characteristics of tumor cells but also linked to the TME. TME consists mainly of immune cells, such as T cells, B cells, and NK cells, and stromal cells, such as fibroblasts and endothelial cells (Domingues et al., 2016). The different immune cells play varied roles in tumor cell proliferation, apoptosis, and metastasis. Therefore, the number and proportion of immune cells in metastatic tissues play an important role in the classification of tumor subtypes (Hanahan and Coussens, 2012; Gambardella et al., 2020). For example, infiltration of CD8^+^ T cells is often associated with a positive prognosis, whereas polarization of M2 macrophages is considered a negative prognostic marker (Petitprez et al., 2020). Numerous studies show that a wide range of proteins in immune and tumor cells undergo extensive neddylation, and high expression levels of many neddylation-associated proteins in tumors were indicative of poor patient outcomes (Chang et al., 2012; Li et al., 2013; Zhou L. et al., 2019). Therefore, by regulating the abundance of immune cells in TME, neddylation modification may provide new insights into the pathogenesis and treatment of LUAD.

## Materials and methods

### Data collection and processing

The LUAD expression profile, clinical information, and mutation data were downloaded from the TCGA (The Cancer Genome Atlas) database. After standardization and data collation, 500 tumor samples were eventually obtained from TCGA for further study. Expression profile and clinical information data from the GSE72094 dataset were obtained from the Gene Expression Omnibus (GEO) database. After standardization and data collation, 398 tumor samples from the GSE72094 dataset were finally obtained. Neddylation-associated genes were obtained from the Reactome database (https://reactome.org/). Genetic mutation data and Copy Number Variation (CNV) data were downloaded from the TCGA-LUAD. Public databases GeneMANIA (https://genemania.org/) and STRING (https://cn.string-db.org/) are used to analyze the protein-protein interactions.

### Unsupervised consensus clustering

In this study, a Univariate Cox analysis of neddylation-associated genes was performed and 76 prognostic genes based on the clinical information and expression data were finally obtained from the TCGA database. Unsupervised consensus clustering analysis based on expression profile data from 76 prognostic genes was carried out using the R package “ConsensusClusterPlus” (Wilkerson and Hayes, 2010). The optimal clustering number was selected based on the Cumulative Distribution Curve. Principal Component Analysis (PCA) further confirmed the validity of clustering. Consensus clustering of differentially expressed core genes between two neddylation patterns used the same approach, and two genomic subtypes were eventually obtained.

### Tumor immune microenvironment

In this study, ESTIMATE (Yoshihara et al., 2013), EPIC (Racle et al., 2017), TIMER (Li et al., 2016), and single-sample Gene Set Enrichment Analysis (ssGSEA) algorithms (Lin et al., 2021) were used to determine the TME. The ESTIMATE algorithm evaluated the ESTIMATE score, immune score, and stromal score, and analyzed tumor purity. The EPIC algorithm was used to demonstrate the infiltration abundance of seven immune cell types in the tumor. The TIMER algorithm was applied to evaluate the infiltration abundance of six immune cell types. Additionally, the ssGSEA algorithm calculated the infiltration abundance of 24 immune cell types (Bindea et al., 2013).

### Functional enrichment analysis and identification of core gene networks

The gene set variation analysis (GSVA) algorithm was used to analyze functional differences between two neddylation patterns (Hanzelmann et al., 2013). Hallmark gene sets were downloaded from the GSEA website (Liberzon et al., 2015). The enrichment score of each sample in the gene set was calculated using the R package “GSVA,” resulting in enrichment score matrix. Mariathasan et al. (2018) compiled nine tumor-associated biological pathways. Differential genes between the two neddylation patterns were obtained using the R package “limma” (Ritchie et al., 2015). Kyoto Encyclopedia of Genes and Genomes (KEGG) and Gene Ontology (GO) databases were analyzed based on differentially expressed genes (FDR < 0.05, |log2FC| > 1) between the two patterns (The Gene Ontology, 2019; Kanehisa et al., 2021). KEGG analysis was performed using the R package “Cluster Profiler” (version 3.14.3). Metascape website was used to carry out GO analysis (Zhou Y. et al., 2019). The protein-protein interaction (PPI) network was constructed using the STRING database and the network connection type to “physical connection” with a confidence score of ≥0.4 was set (Szklarczyk et al., 2021). In addition, to build the network, Cytoscape software was used to calculate Degree scores and screen for core genes (Degree > 10) (Shannon et al., 2003).

### Construction of neddylation score

In this study, R package “glmnet” was used to perform LASSO-Cox analysis (10-fold cross-validation). Multivariate stepwise regression was then performed on 17 genes obtained from LASSO. Finally, a prognostic signature consisting of 10 genes was developed. The formula for the signature was computed as follows: risk score = [Coef(1) × gene Exp(1)] + [Coef(2) × gene Exp(2)] + …… + [Coef(i) × gene Exp(i)] (Tibshirani, 1997; Wang et al., 2019). Prognostic analysis was performed by Kaplan-Meier curve using R packets “survival” and “survminer.” The R packages “timeROC” and “survival” were used to assess 1-, 3-, and 5-year survival.

### Prediction of chemotherapeutic drug sensitivity

R package “pRRophetic” was used to predict the efficacy of chemotherapy drugs (Geeleher et al., 2014). Minimum drug inhibition concentrations (IC_50_) were calculated for each sample based on expression profile data from patients with LUAD. By comparing chemotherapeutic drug sensitivity between high- and low-risk groups, better personalization of LUAD treatment was possible by selecting specific drugs.

## Results

### Prognostic value and genetic variation of neddylation-associated genes in lung adenocarcinoma

**FIGURE 1:**
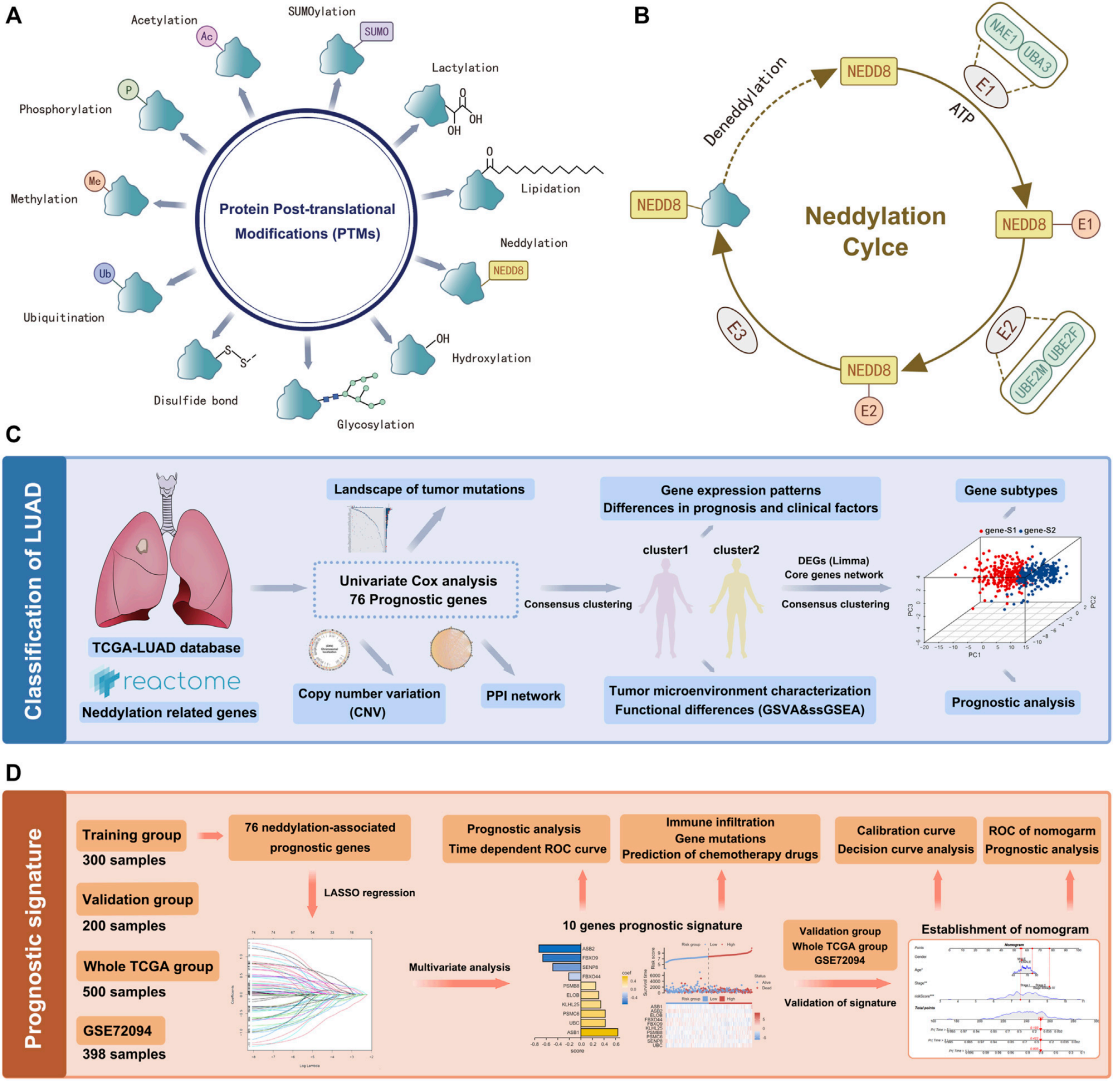
Flow chart of the data analysis. **(A)** PTMs of proteins. **(B)** Process of neddylation modification. **(C)** LUAD classification based on neddylation-related genes. **(D)** Prognosis signature based on neddylation-related genes.

**FIGURE 2:**
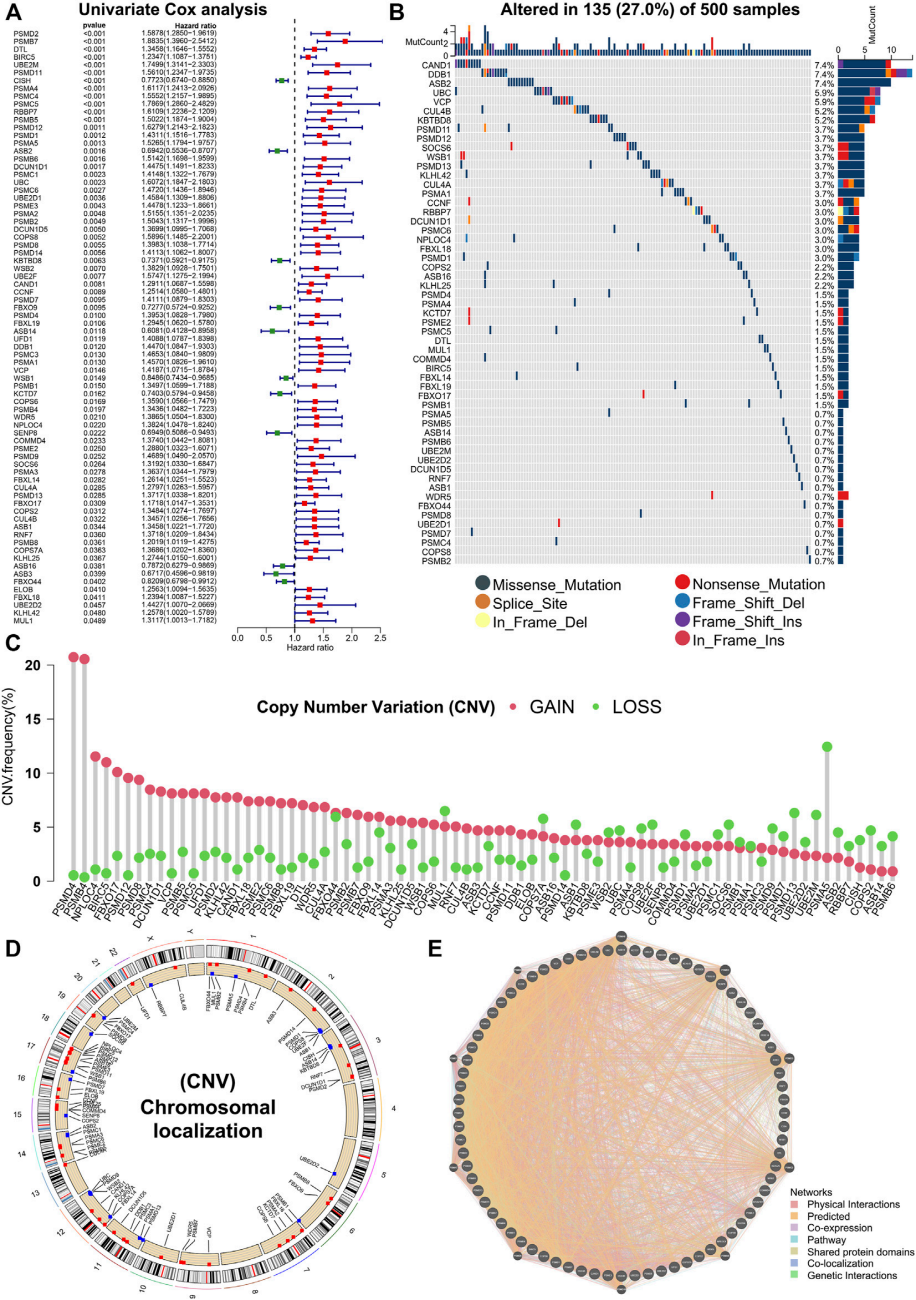
Genetic variants in 76 prognostic genes. **(A)** Univariate Cox analysis identifying 76 prognostic genes. **(B)** Mutation landscape of prognostic genes. **(C,D)** CNV in prognostic genes. **(E)** PPI network of prognostic genes.

Various types of PTMs include hydroxylation, lipidation, glycosylation, disulfide bond, ubiquitination, methylation, phosphorylation, acetylation, SUMOylation, lactylation, neddylation, etc. (Figure 1A). Studies have shown that protein PTMs play a crucial role in many biological processes in cancer malignancy. Neddylation is an important PTM, and a reversible process regulated by NEDD8, neddylation E1 activating enzymes (NAE1, UBA3), neddylation E2 binding enzymes (UBE2M, UBE2F), neddylation E3 ligases, de-neddylation proteins, etc. (Figure 1B). Neddylation affects the stability, conformation, and function of substrate proteins, which in turn regulate nuclear localization, intracellular signaling, DNA damage response, cell cycle, apoptosis, and the TME. Based on the role of neddylation in tumor progression, the gene set from the Reactome database was obtained, and neddylation-associated proteins were selected for further study. Figures 1C,D showed the flowchart of our study in LUAD. In this study, a Univariate Cox analysis of neddylation-associated genes was performed and 76 genes with prognostic values were identified (Figure 2A). To further explore the value of these 76 genes in tumors, their mutational status in LUAD was analyzed. A total of 135 samples were found to have mutations in these genes in 500 tumor samples, with an overall incidence of 27%, mainly in the form of missense mutations and nonsense mutations (Figure 2B). In addition, CNV analysis of the prognostic genes was also conducted. CNV amplifications were prevalent in many genes, especially PMD4 and PMB4, whereas CNV deletions mainly existed in PMA5, PMD13, and UBE2M (Figure 2C). CNV occurred on many chromosomes but was mainly concentrated on chromosomes 1, 2, 3, 7, 11, 12, 14, 15, 17, and 19 (Figure 2D). A PPI network of neddylation-related prognostic genes was also constructed. The protein network consisted of seven types of connections: physical interactions, predicted, co-expression, pathway, shared protein domains, co-localization, and genetic interactions. The neddylation-associated genes were found to have a common interaction and were predicted to play a synergistic role in tumors. To further explore the function of 76 prognostic genes, an enrichment analysis was performed using metascape (Supplementary Figures S1A,B). The results showed that these genes play an important role in biological processes such as neddylation, antigen processing, protein modification, and negative regulation of the immune system process.

### Identification of two different molecular patterns of lung adenocarcinoma based on neddylation-related genes

**FIGURE 3:**
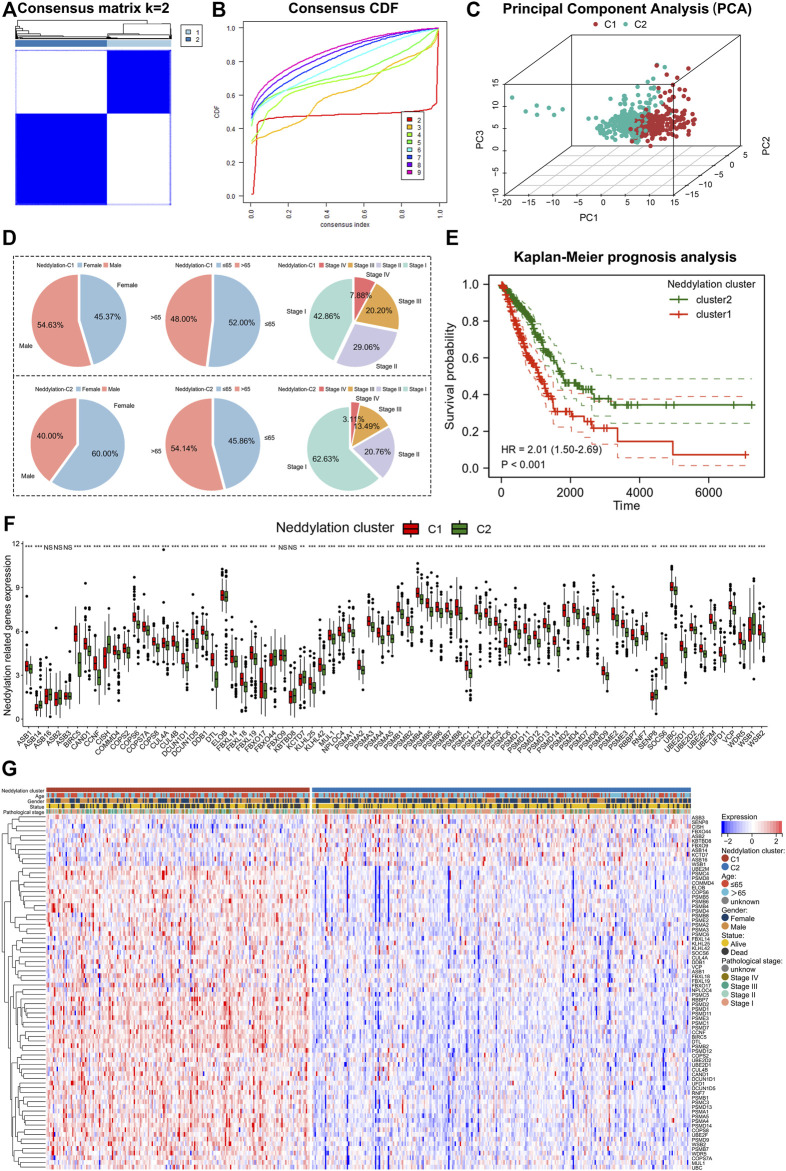
Identification of two patterns associated with neddylation. **(A,B)** Consensus clustering of 76 genes in LUAD. **(C)** PCA in two patterns. **(D)** Differences in clinical factors between the two patterns. **(E)** Differences in prognosis between the two patterns. **(F,G)** Expression of 76 prognostic-related genes in two patterns.

Based on consensus clustering, the TCGA obtained LUAD samples were categorized into two patterns using neddylation-associated prognostic genes (Figure 3A). The highest stability between the two patterns existed when *k* = 2 (Figure 3B). Principal component analysis (PCA) further validated the significant differences between the two patterns (Figure 3C). The two patterns were labeled as neddylation cluster1 and neddylation cluster2, respectively. The two patterns differed in clinicopathologic factors in patients with LUAD (Figure 3D). Cluster1 had more male patients and also more patients aged ≤65 years compared with cluster2. Considering pathological stage, cluster1 had more patients with pathological stage III and stage IV tumors compared with cluster2. Cluster1 also demonstrated a worse prognosis than cluster2 using patient outcomes (Figure 3E). The expression of 76 prognostic genes between the two patterns was explored. The vast majority of neddylation-related genes are differentially expressed in both patterns (Figures 3F,G). Therefore, further exploring the two patterns of LUAD is of practical significance.

### Immune landscape and functional differences between the two patterns

**FIGURE 4:**
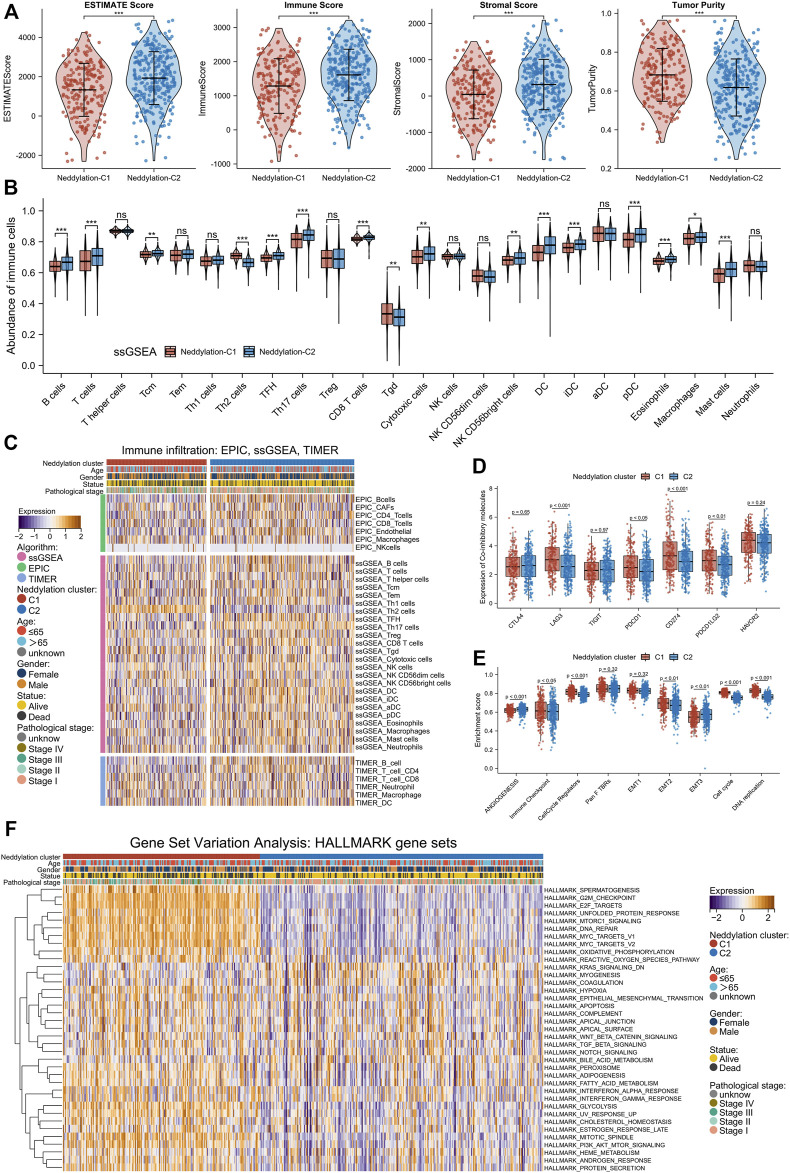
Two patterns revealed differences in TME and biological function. **(A)** ESTIMATE score, immune score, stromal score, and tumor purity of the two patterns. **(B)** Abundance of infiltration of 24 immune cells calculated by ssGSEA algorithm. **(C)** Differences in immune infiltration abundance between different patterns and clinical factors (EPIC, TIMER, ssGSEA algorithm). **(D)** Differences in expression of immune co-inhibitors between the two subtypes. **(E)** Calculation of enrichment scores of nine gene sets using ssGSEA algorithm. **(F)** Statistical differential pathways obtained by GSVA algorithm.

The immune infiltration abundance of TCGA samples was calculated using the ESTIMATE algorithm. By comparing the scores of cluster1 and cluster2, cluster1 was found to have higher tumor purity but conversely lower ESTIMATE, immune, and stromal scores (Figure 4A). These results were further validated by using ssGSEA, TIMER, and EPIC algorithms: results of the ssGSEA algorithm, which calculated enrichment scores for 24 immune cell types, revealed that cluster1 generally had a lower abundance of immune cells such as B cells, T cells, CD8^+^ T cells, cytotoxic cells, dendritic cells (DC), and mast cells (Figure 4B); the TIMER and EPIC algorithms calculated enrichment scores for six and seven immune cell types, respectively, and results from both indicated that cluster1 had a lower abundance of CD4^+^ T cells and B cells (Figure 4C). In short, these four algorithms suggested that cluster1 had lower levels of immune infiltration and was favorable for tumor escape. These findings were consistent with the prognostic results between the two patterns. Besides, immunotherapies targeting immune co-suppressor molecules have become a very important topic in the clinical treatment of lung cancer, particularly in treating adenocarcinoma. The differences in immune checkpoint expression levels between the two patterns were compared. As shown in Figure 4D, cluster1 had higher expression levels of LAG3, PDCD1, CD274, and PDCD1LG2 compared to cluster2, suggesting that patients in cluster1 may benefit from immunotherapies. To further investigate the differences in the functional mechanism between the two patterns, the ssGSEA method was used to compute nine gene sets reported by Mariathasan et al. (Angiogenesis, Immune checkpoint, Cell cycle regulators, Pan F TBRs, EMT1, EMT2, EMT3, Cell cycle, DNA replication). Results showed that cluster1 had higher levels of immune checkpoints, cell cycle regulators, EMT2, cell cycle, and DNA replication (Figure 4E). This meant that patients with LUAD in cluster1 had higher expression of immune checkpoints and also enhanced biological functions associated with cell proliferation and metastasis. In addition, the GSVA analysis was used to calculate scores for 50 gene sets from the Hallmark pathway and ultimately found statistical differences in 37 pathways between the two patterns (Figure 4F). Notably, cluster1 was significantly enriched at G2M checkpoints, DNA repair, E2F targets, MTORC1 signaling, MYC targets, Glycolysis, EMT, TGF-β signaling, PI3K-AKT-MTOR signaling, and many other tumor progression–related pathways (Supplementary Figure S2). Combining the results of ssGSEA and GSVA algorithms, cluster1 was found to exhibit a pro-tumor progression pattern in molecular mechanisms, which better explained the worse prognosis observed in the patients of this pattern.

### Construction of two genomic subtypes based on differentially expressed genes

**FIGURE 5:**
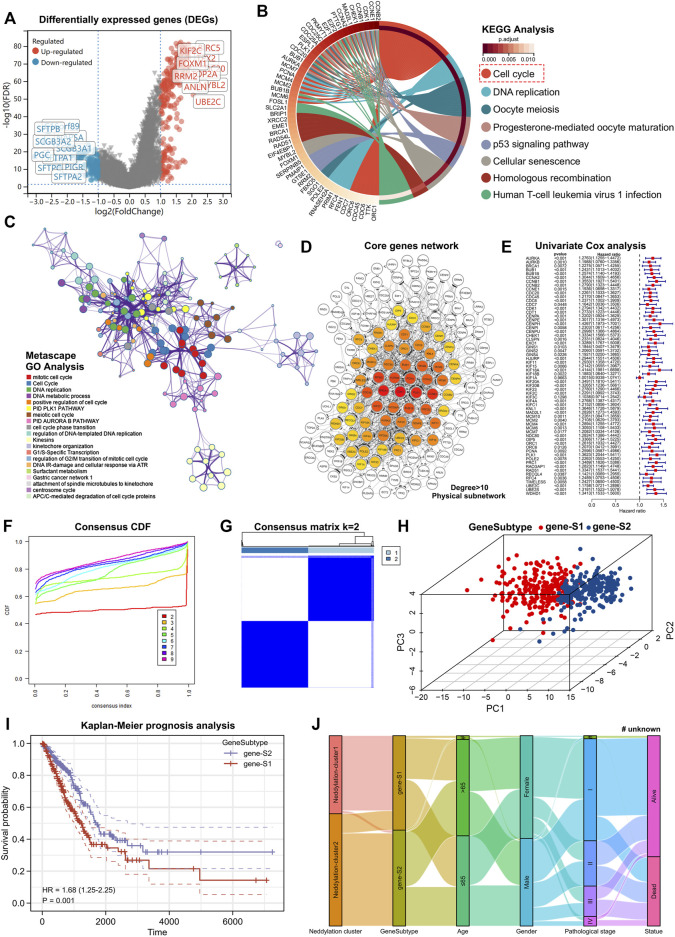
Identification of neddylation-associated gene subtypes based on differentially expressed genes. **(A)** Volcano map showed differentially expressed genes between two patterns (FDR < 0.05, |log2FC| > 1). **(B)** KEGG enrichment analysis of differentially expressed genes (FDR < 0.05, |log2FC| > 1). **(C)** GO enrichment analysis of differentially expressed genes (FDR < 0.05, |log2FC| > 1). **(D)** Construction of core gene network using STRING database and cytoscope software. **(E)** Univariate Cox analysis of core genes. **(F,G)** Consensus clustering based on core genes. **(H)** PCA of gene subtypes. **(I)** Prognostic differences between two gene subtypes. **(J)** Sankey diagram based on neddylation patterns, gene subtypes, age, gender, pathological stage, and survival status.

Using the R package “limma,” differentially expressed genes between the two patterns (|log2FC| > 1, FDR < 0.05) were identified. The red dots on the volcanic map were the upregulated genes and the blue dots were the downregulated genes (Figure 5A). Based on differentially expressed genes, KEGG and GO analyses were performed. Eight pathways were observed to be enriched by KEGG Figure 5B. Substantial enrichment of the cell cycle–related pathways was observed (Supplementary Table S1). Additionally, GO analysis was performed using metascape and enriched modules were exhibited in different color regions (Figure 5C). In these modules, many biological functions such as cell cycle, DNA replication, and cell cycle checkpoints were found to be involved; these results were consistent with the KEGG analysis. To further investigate the core genes that play a central role in these differentially expressed genes, constructed a core PPI network was constructed using the STRING database (network type: physical subnetwork, the minimum required interaction score: 0.4) and Cytoscape software (Cytohubba plugin, Degree > 10). Eventually, a core network of 62 genes was obtained (Figure 5D). A Univariate Cox analysis was performed on 62 core genes and all the core genes were found to be related to prognosis (Figure 5E). To further investigate the overall role of core genes, an unsupervised consensus clustering was used to classify the patients with LUAD obtained from the TCGA. Notably, patients with LUAD can be classified into two genomic subtypes, called genesubtype-S1 and genesubtype-S2 (Figures 5F,G). Results from the PCA showed significant differences between the two genomic subtypes (Figure 5H). It was observed that the prognosis of patients in genesubtype-S1 was worse (Figure 5I). A Sankey diagram was drawn to better understand the direct relationship between neddylation patterns and genomic subtypes and observed that the vast majority of neddylation cluster1 and a small fraction of neddylation cluster2 made up genesubtype-S1 (Figure 5J). Whereas the vast majority of neddylation cluster2 constituted genesubtype-S2. These results suggested that differential expressed genes obtained from neddylation patterns can identify two genomic subtypes with underlying differences in biological functions.

### Construction of neddylation-associated prognostic signature

**FIGURE 6:**
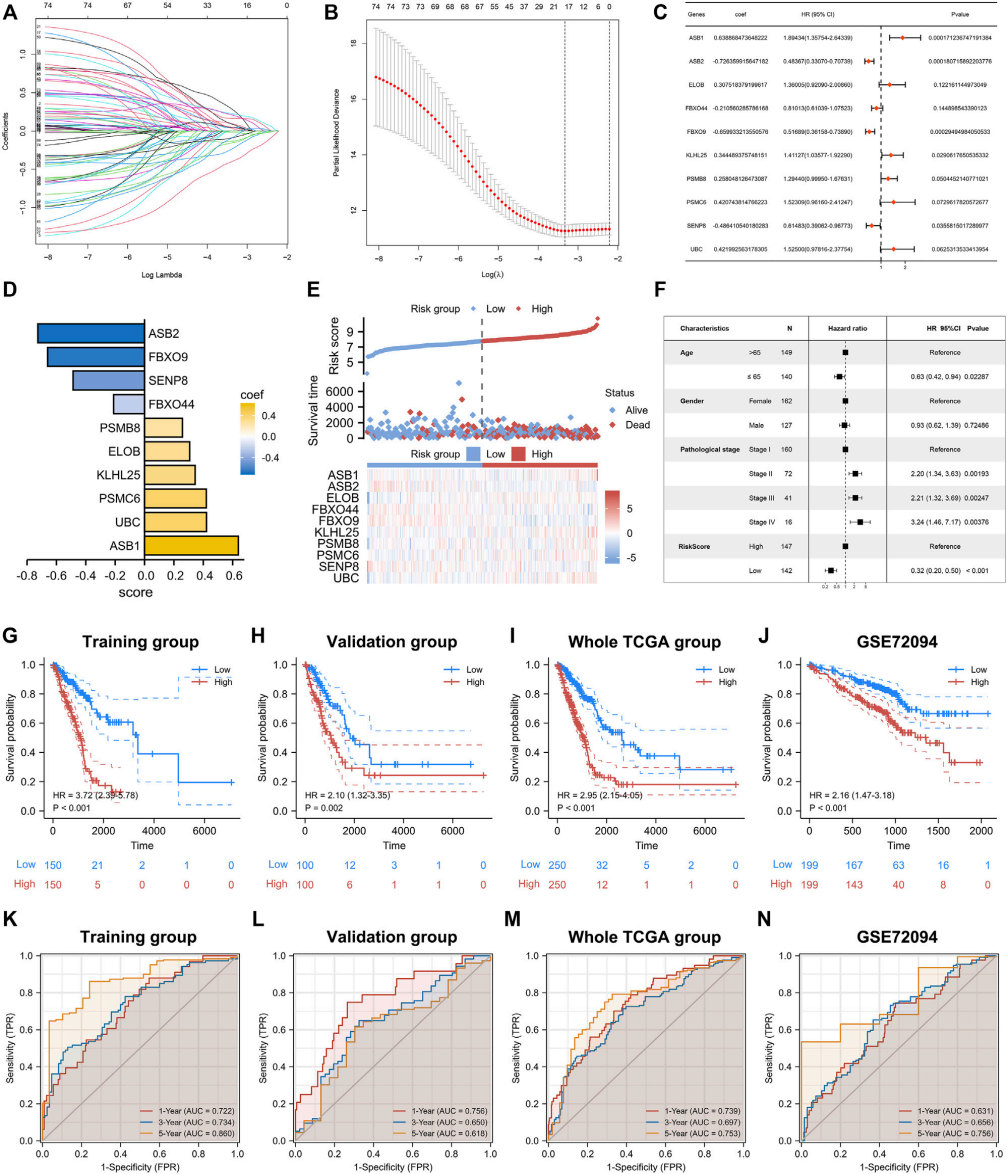
Construction of prognostic signature. **(A,B)** LASSO regression analysis based on 76 prognostic genes. **(C)** Multivariate Cox analysis was performed on genes obtained from LASSO. **(D)** The 10 genes that ultimately built the signature. **(E)** Differences in survival status and expression of 10 genes between high- and low-risk groups. **(F)** Multivariate Cox analysis of risk score and clinical factors. **(G–J)** Prognostic analysis of training set, validation set, whole TCGA Set, GSE72094. **(K–N)** Time-dependent ROC analysis of the training set, validation set, whole TCGA set, and GSE72094.

Based on the above analyses, we reconfirmed the biological functions of neddylation-associated genes in LUAD. Therefore, constructing a neddylation-associated prognostic signature was relevant for more accurate risk stratification and personalized treatment. First, conducted a LASSO-Cox regression analysis was conducted of the 76 prognostic genes to rule out co-linearity (Figures 6A,B). The obtained genes were then subjected to Multivariate stepwise regression analysis and 10 genes were ultimately obtained (Figures 6C,D). Based on the coefficients of these 10 genes, the following formula was computed: risk score = 0.638868474 * (ASB1 expression) + (−0.726359916) * (ASB2 expression) + 0.307518379 * (ELOB expression) + (−0.210560286) * (FBXO44 expression) + (−0.659933214) * (FBXO9 expression) + 0.344489376 * (KLHL25 expression) + 0.258048126 * (PSMB8 expression) + 0.420743815 * (PSMC6 expression) + (−0.48641054) * (SENP8 expression) + 0.421992563 * (UBC expression). Based on this formula, LUAD samples from the training set were classified into high- and low-risk groups. The heat map showed the expression of 10 genes in the high- and low-risk groups and a notably higher death rate was observed in the high-risk group (Figure 6E). Furthermore, a Multivariate Cox analysis was performed for patients with LUAD by risk score, age, gender, and pathological stage. The results showed that risk score can be an independent prognostic factor (Figure 6F). In addition, using the same formula, risk scores in the validation set, the entire TCGA database, and the external validation dataset GSE72094, were calculated. Prognostic analysis of four databases was conducted and significantly worse outcomes in all datasets for the high-risk group were observed Figures 6G–J. Additionally, a time-dependent receiver operating characteristic (ROC) analysis was performed on these four datasets to validate the predictive efficiency of signatures. The survival rates in the training set of 1, 3, and 5 years were 0.722, 0.734, and 0.860, respectively (Figure 6K). The validation sets also showed good predictive performance (Figures 6L–N). These results confirmed the accuracy of the 10-gene signature in determining patient risk stratification and prognosis. Moreover, to better investigate the relationship between risk scores and clinical parameters, a more refined examination was conducted. The results showed no statistically significant difference in risk scores between patients aged ≥65 years compared to those aged <65 years. However, in terms of gender, male patients had significantly higher risk scores than female patients. We also found that patients with pathological stage III and IV had higher risk scores than patients with pathological stage I and II (Supplementary Figures S3A–C). In addition, we performed prognostic analyses for patients with different clinical parameters. Excitingly, the results revealed that patients with high-risk scores in these clinical parameters all had a poorer prognosis (Supplementary Figures S3D–I).

### Predicting immune infiltration, genetic mutations, and chemotherapeutic drug efficacy based on the risk score

**FIGURE 7:**
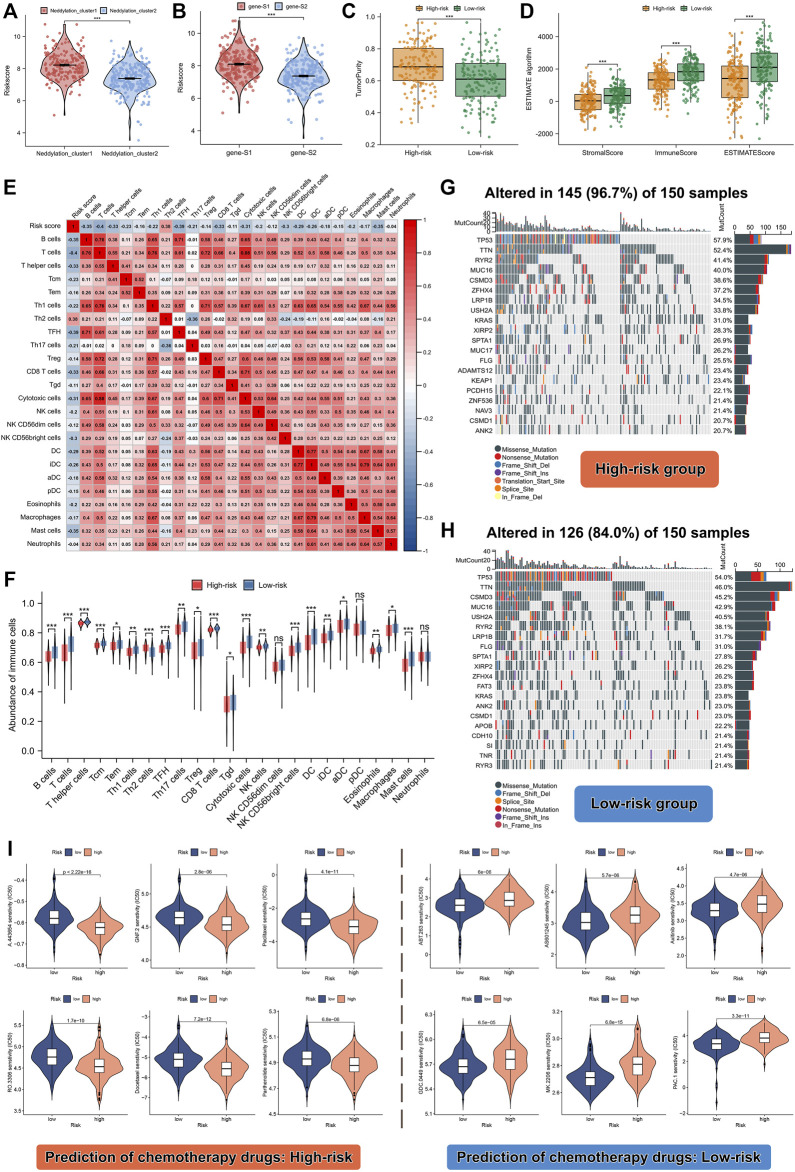
Differences in TME, mutation levels, and chemotherapeutic drug sensitivity among high- and low-risk patients. **(A)** Risk score between the two different neddylation patterns. **(B)** Risk score between the two different gene subtypes. **(C,D)** Tumor purity, estimate score, immune score, and stromal score between high- and low-risk groups. **(E,F)** Correlation between risk score and abundance of 24 immune cell types. **(G,H)** Correlation between risk score and genetic mutation. **(I)** Prediction of chemotherapeutic drugs in high- and low-risk groups.

As discussed earlier, two molecular patterns and two genomic subtypes associated with neddylation were identified. We further analyzed the relationship between risk scores and different subtypes. As shown in Figures 7A,B, neddylation cluster1 and genesubtype-S1 had a higher risk score; these findings were consistent with previous analysis. After confirming the prognostic efficacy of the signature, we further explored whether risk score can be used to determine immune infiltration, gene mutation status, and chemotherapeutic drug selection in patients with LUAD. The ESTIMATE algorithm helped identify the high-risk group with higher tumor purity and lower ESTIMATE, immune, and stromal scores. The high-risk group exhibited lower levels of immune infiltration (Figures 7C,D). To validate these results, ssGSEA analysis was performed on the training set. Interestingly, except for Th2 cells, the majority of immune cells exhibited a low abundance of immune infiltration (Figures 7E,F). These results suggested that the high-risk group had a negative TME that promotes tumor progression. Remarkably, the high-risk group also displayed notable differences in the extent of mutations compared to the low-risk group: mutations occurred in 145 of the 150 samples in the high-risk group, versus in 126 of the 150 samples in the low-risk group. Additionally, heat maps identified the 20 genes with the highest mutation rates in both groups, and significant differences were observed in the frequency of mutations in these genes (Figures 7G,H). Chemotherapeutic drug predictions were also performed for patients with LUAD in the high- and low-risk groups to provide options for personalized treatment. The high-risk group responded better with A.443654, GNF.2, Paclitaxel, Parthenolide, RO.3306, and Docetaxel, whereas the low-risk group responded better with ABT.263, AS601245, Axitinib, GDC.0449, MK.2206, PAC.1 (Figure 7I).

### Building a more accurate nomogram that combines clinical factors with risk score

**FIGURE 8:**
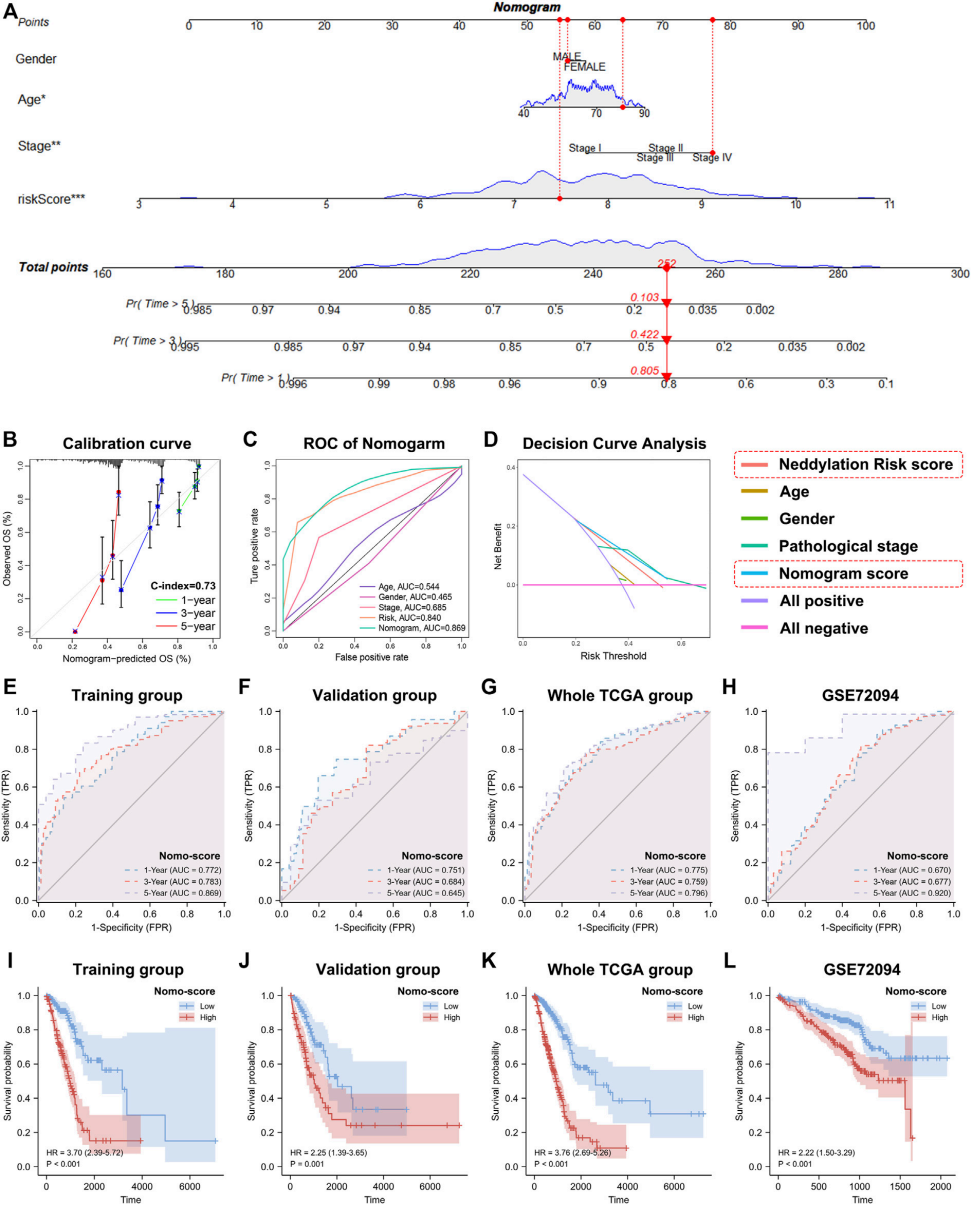
Construction of nomogram based on risk score and clinical factors. **(A)** A more predictive nomogram. **(B)** Calibration curve of 1-year, 3-year, and 5-year survival time. **(C)** Nomogram’s 5-year survival time ROC curve. **(D)** Nomogram’s 5-year survival time DCA curve. **(E–H)** Time-dependent ROC curves of the training set, validation set, entire TCGA set, and GSE72094 (based on nomo-score). **(I–L)** Kaplan-Meier analyses of the training set, validation set, entire TCGA set, and GSE72094 (based on nomo-score).

To improve the accuracy of the predictive effect of the risk score, the risk score was combined with clinical factors to construct a nomogram (Figure 8A). By establishing a calibration curve, the nomogram demonstrated good accuracy in predicting the 1-year, 3-year, and 5-year survival times (Figure 8B). The decision curve analysis (DCA) and ROC curves for 5-year survival revealed that nomo-scores and risk scores were better predictors for survival than in the pathological stage (Figures 8C,D). These findings confirmed that our signature has good prospects in clinical application. Additionally, we also constructed nomograms for the validation set, the entire TCGA dataset, and the external validation set GSE72094 to further confirm the validity of our nomogram and validated the prognosis accuracy across the four datasets (Figures 8E–H). The results revealed that the prognosis of patients with LUAD could be significantly distinguished based on the nomo-score: patients with a high nomo-score had a worse prognosis. Time-dependent ROC curves were also used to further validate the predictive efficiency of the nomogram and find that the nomo-score has higher AUC values than the risk score (Figures 8I–L). Taken together, these results further confirmed that risk score can be used not only as a prognostic factor alone but also in combination with other clinical factors to substantially improve the accuracy of prognosis determination in patients with LUAD.

## Discussion

Cancer is an extremely complex entity. Many studies have shown that multiple factors are involved and controlled in tumorigenesis. Sustained growth signaling, unlimited replication potential, antiapoptotic factors, acquisition of invasive metastasis, reprogramming of cell metabolism, and TME are major hallmarks of cancer malignancy (Hanahan and Weinberg, 2011; Wong, 2011; Mossmann et al., 2018; Xiao and Yu, 2021; Yin et al., 2022). However, tumors exhibit extreme heterogeneity, which is characterized by mutations in genes and alterations in the TME (Paul et al., 2019; Zhang et al., 2022). Therefore, finely tailored therapeutic strategies are required for tumor treatment.

Neddylation is a type of protein PTM that increases proteomic diversity. Increasing evidence suggests that neddylation is aberrantly activated in a wide range of tumor types, consequently affecting tumor progression and altering the TME (Xie et al., 2021; Zhou et al., 2021). Further research on neddylation-related genes is warranted to provide new treatment strategies for cancer treatment (Zheng et al., 2021). Lung cancer is one of the leading causes of human death worldwide and has a high incidence and mortality rate. LUAD is the most common histological type of lung cancer and has a highly complex TME. Few studies have been conducted on neddylation modification in LUAD, and most of the existing literature focuses on the mechanisms and effects of one particular gene in the neddylation modifications (Zhou et al., 2017; Lee et al., 2018). Therefore, systematically investigating the role of neddylation-associated genes in predicting the prognosis of tumor progression and TME is of practical significance. Furthermore, using neddylation-associated tumor classification and risk stratification, determining the prognosis of LUAD and providing personalized treatment options might be possible.

In this study, 76 neddylation-associated prognostic genes in LUAD were first identified and their mutation frequency and CNV were analyzed. Based on prognostic genes, patients with LUAD were successfully classified into two patterns, viz., cluster1 and cluster2. Cluster1, when compared with cluster2, exhibited a higher proportion of patients with pathological stage III and IV LUAD, thus presenting a more pronounced malignant state with a worse prognosis. Using four immune infiltrating algorithms (ESTIMATE, EPIC, ssGSEA, and TIMER), it was noted that the levels of numerous immune cell types, including B cells, DC, mast cells, CD4^+^ T cells, and CD8^+^ T cells, were significantly lower in cluster1. Based on these results, we can conclude that the immune state in cluster1 promotes tumor progression and tumor escape, whereas, in cluster2, it showed inhibition of tumor progression. However, high levels of immune co-inhibitory molecules, such as LAG3, PDCD1, CD274, and PDCD1LG2, were also expressed in cluster1. This may be suggestive of a better response to immunotherapy among patients with LUAD in cluster1 (Schnell et al., 2020). To further investigate the potential mechanism of action of these two patterns in biological function, the ssGSEA algorithm was used to analyze the nine gene sets identified by Mariathasan et al. as well as Hallmark’s 50 gene sets from the GSEA website. The ssGSEA algorithm analysis, and later confirmed by GSVA, showed that cluster1 demonstrated a tendency to favor tumor proliferation and metastasis, as evidenced by the higher enrichment scores for gene sets of cell cycle regulators, cell cycle, DNA replication, EMT2, immune checkpoints, etc. Cluster1 pattern shows activity across a wide range of pathways and demonstrated high enrichment scores for G2M checkpoints, E2F targets, unfolded protein responses, MYC targets, oxidative photography, mitotic spindle, DNA replay, glycolysis, and other tumor progression–associated biological functions. Notably, the cluster1 pattern was significantly enriched in the following signaling pathways: TNF-α, TGF-β, IL6-JAK-STAT3, PI3K-AKT-mTOR, and MTORC1. Numerous studies have shown that aberrant activation of these signaling pathways substantially contributes to growth, migration, and invasion of tumor cells, thus reshaping the TME and exhibiting worse prognosis (Kim et al., 2017; Johnson et al., 2018; Saito et al., 2018; Cruceriu et al., 2020; Iksen et al., 2021). Additionally, cluster1 also demonstrated notably increased adipogenesis, fatty acid metabolism, and cholesterol homeostasis; abnormal levels of metabolism play an important role in tumor progression as has been confirmed by previous studies (Li and Zhang, 2016; Ghaben and Scherer, 2019; Liu et al., 2021).

As there were differences in biological functions across the two neddylation patterns, differential expression analysis was performed which helped to further identify cell cycle-related genes that may play a central role in tumor progression and metastasis. Building and analysis of the core network helped to identify the 62 most critical genes. Additionally, through an unsupervised consensus clustering approach, LUAD was subdivided into two genomic subtypes, viz., genesubtype-S1 and genesubtype-S2. Genesubtype-S1 demonstrated a worse prognosis compared with genesubtype-S2, again demonstrating the importance of neddylation-associated genes in classifying patients with LUAD. Furthermore, for accurate risk stratification of patients with LUAD, a neddylation-associated prognostic signature was constructed. The signature was highly predictive: considerably worse outcomes were observed in patients with a high-risk score in the training set, validation set, whole TCGA set, and GSE72094. The ROC analysis also showed high predictive accuracy. Notably, most immune cell types had low infiltration abundance in patients with LUAD with high-risk scores, suggesting tumorigenesis and tumor progression were favored in these patients. Genetic mutation analysis revealed that patients with high-risk scores had higher mutation frequency versus those with the low-risk score. Increased genetic mutations may lead to cellular physiological dysfunction and enhanced tumor metastasis. Finally, the sensitivity to chemotherapeutic agents was analyzed and appropriate chemotherapeutic agents were screened for both high- and low-risk groups of patients with LUAD to provide for a more refined treatment strategy.

Many LUAD-associated prognostic models have been made available previously; for example, an immune-related prognostic signature constructed by Yi et al. (2021), a ferroptosis-associated prognostic signature introduced by Wang et al. (2021), an autophagy-related prognostic signature developed by Li et al. (2022), and an inflammatory-associated prognostic signature identified by Zhai et al. (2021) have identified. Compared with these models, our study reported a prognostic model based on neddylation-associated genes which shows promising prognostic accuracy. To our knowledge, this study is the first of its kind. The 10-gene prognostic model constructed in this study aids in determining prognosis. Risk-score outperforms pathological stage in terms of predicting prognosis accurately. As an independent prognostic factor and clinically useful indicator, a risk score can be combined with other clinical factors to construct nomogram with increased accuracy and potential clinical application. However, this study has limitations. Our analysis was based primarily on TCGA and GEO databases, and further validation of the accuracy of our model in larger sample sizes observed in clinical studies would be required.

## Conclusion

In summary, this study aimed to classify patients with LUAD into two patterns based on their neddylation-associated prognostic genes which potentially indicates the nature of TME. Significant differences were observed between the two patterns in the activation of the signaling pathways associated with tumor proliferation and metastasis. Prognostic signatures based on neddylation-related genes can help stratify patients with LUAD, guide personalized treatment, and provide a preliminary exploration for clinical use.

## Data Availability

The datasets presented in this study can be found in online repositories. The names of the repository/repositories and accession number(s) can be found in the article/Supplementary Material.
