# Supplementary figures and images for: Neddylation pattern indicates tumor microenvironment characterization and predicts prognosis in lung adenocarcinoma
Source: Front Cell Dev Biol. 2022 Sep 13;10:979262. doi: 10.3389/fcell.2022.979262 (PMC9513323; doi:10.3389/fcell.2022.979262)

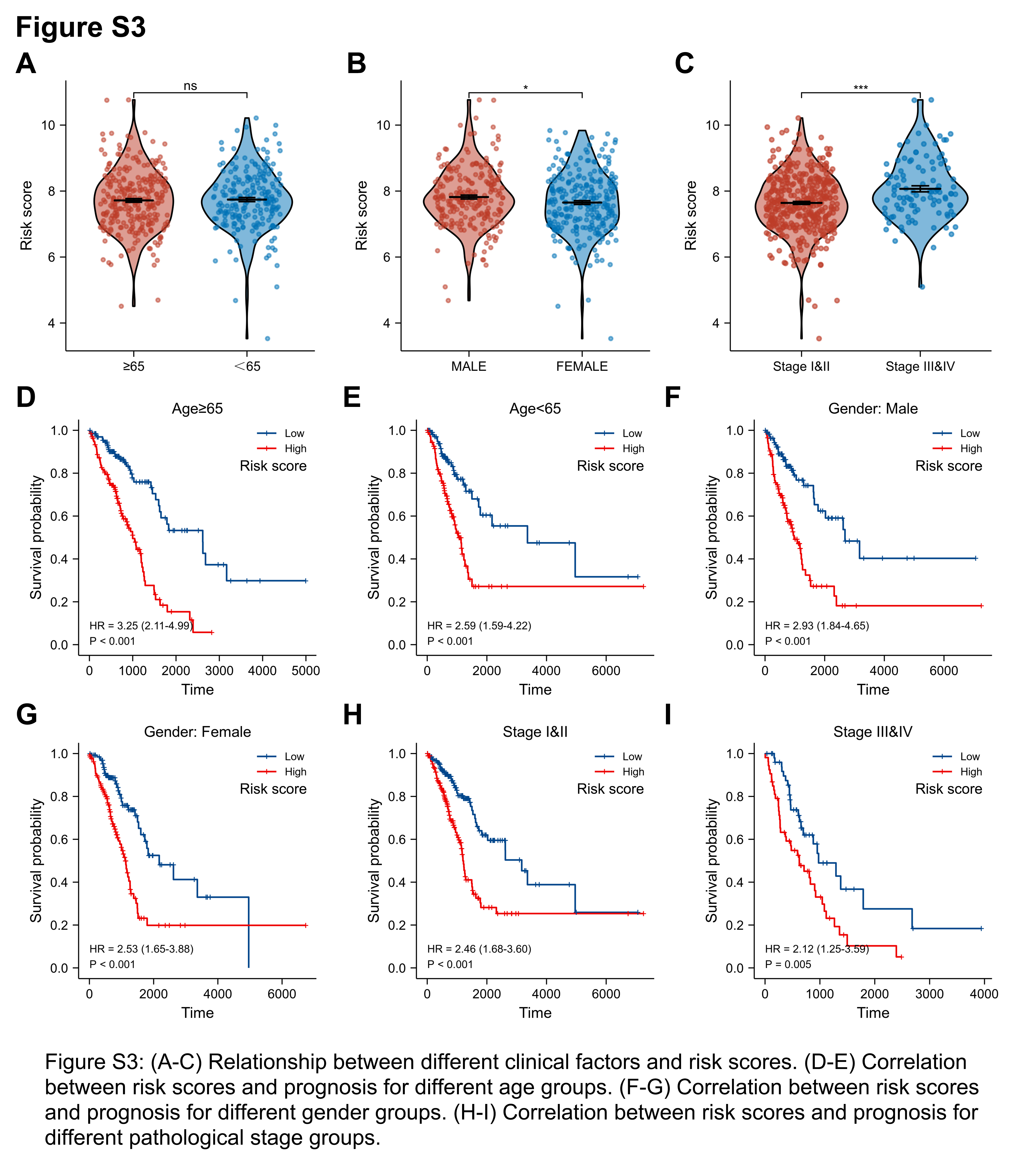

Supplement: Supplementary file 1 [file Image3.JPEG]

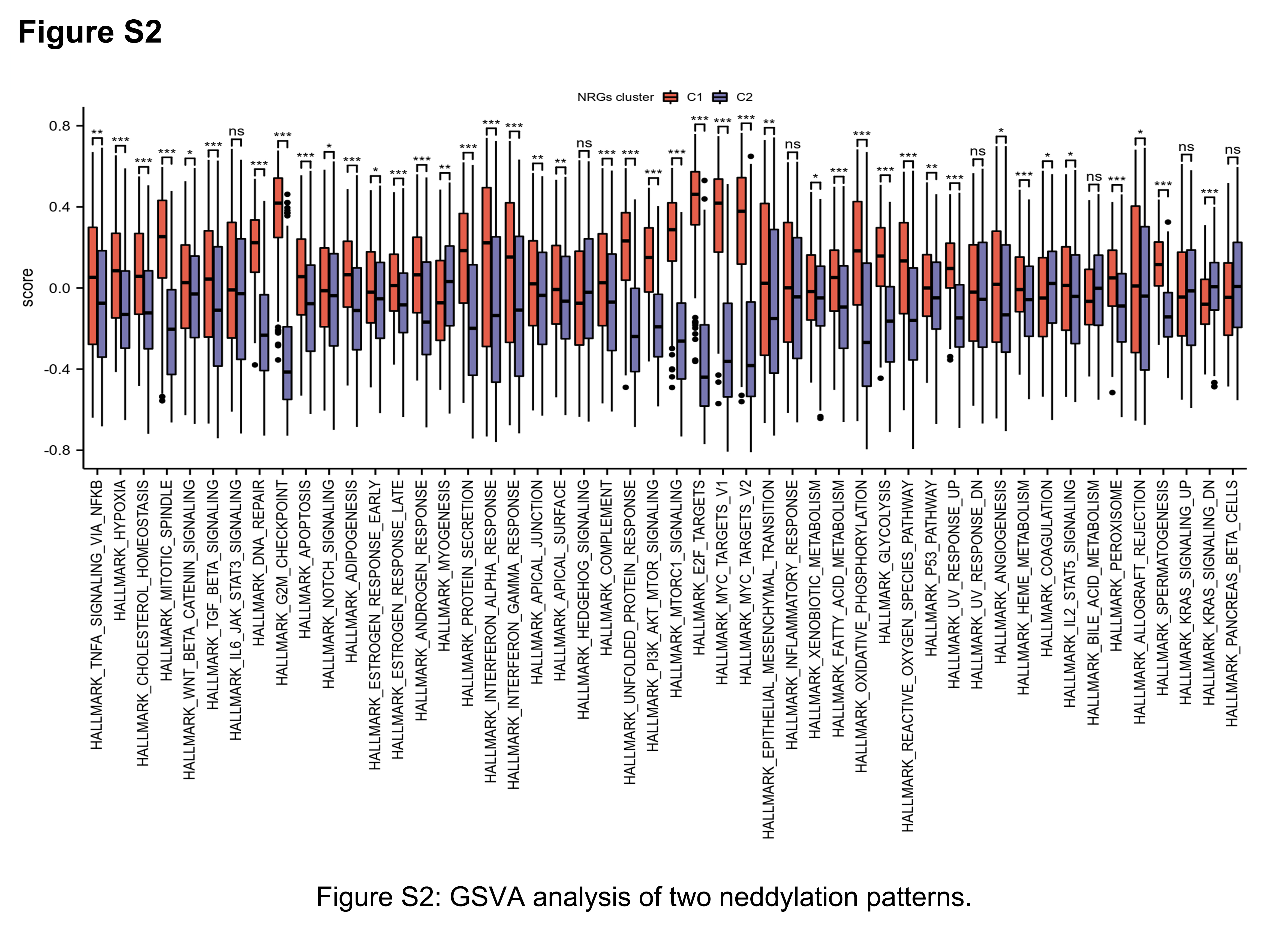

Supplement: Supplementary file 2 [file Image2.jpg]

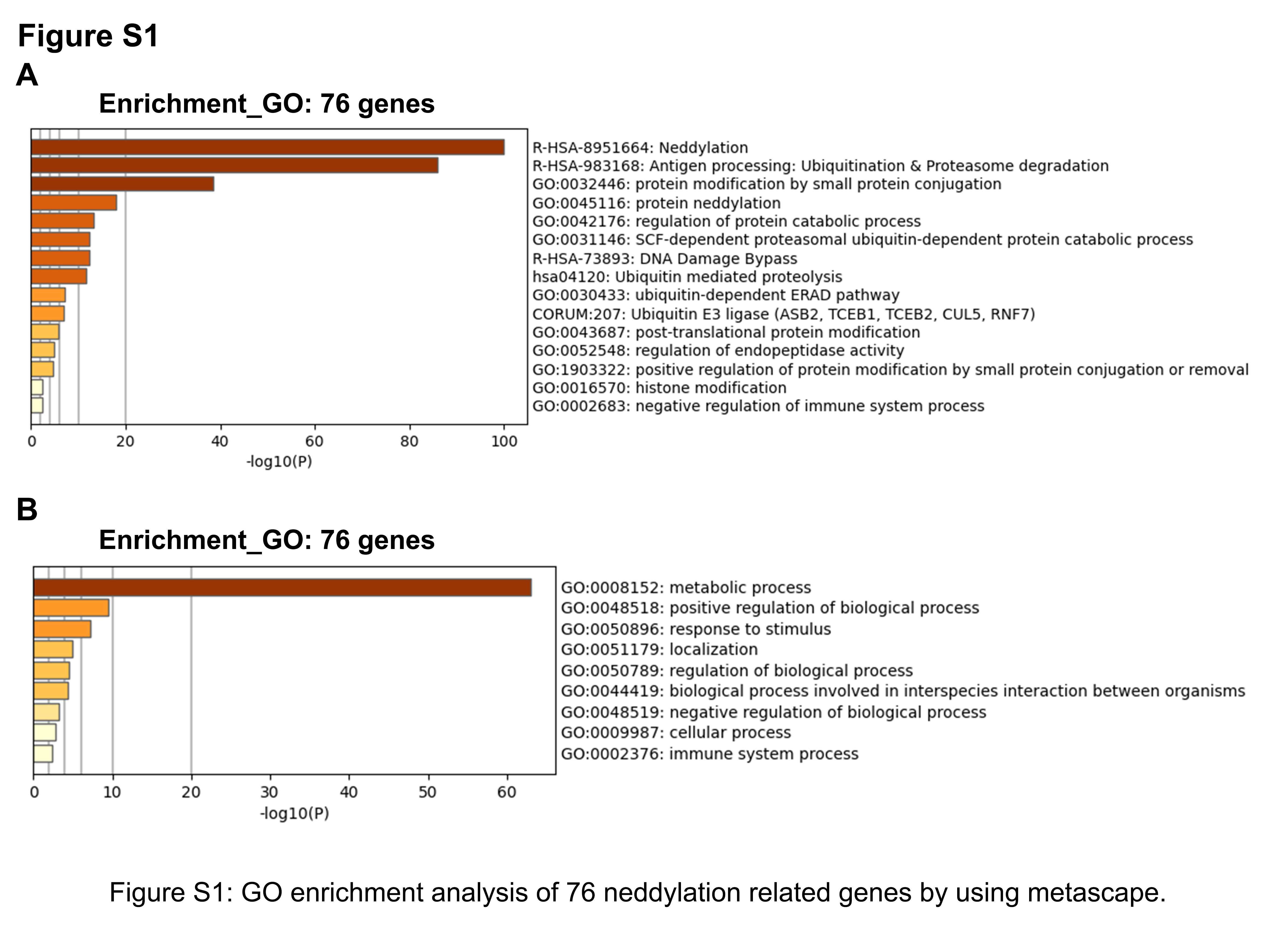

Supplement: Supplementary file 3 [file Image1.JPEG]
